# Awareness regarding risk factors and determinants of cancers among Bahir Dar city residents, Northwest Ethiopia

**DOI:** 10.1371/journal.pone.0248520

**Published:** 2021-04-23

**Authors:** Getasew Mulat Bantie, Amare Alamirew Aynie, Yared Mulu Gelaw, Ayele Semachew Kasa, Meron Asmamaw Alemayehu, Koku Sisay Tamirat, Gebiyaw Wudie Tsegaye, Gizachew Tadesse Wassie, Tigabu Birhan Kassa, Amanuel Addisu Dessie

**Affiliations:** 1 Faculty of community Health, Alkan Health Sciences Business and Technology College, Bahir Dar, Ethiopia; 2 Faculty of Medical Health, Alkan Health Sciences Business and Technology College, Bahir Dar, Ethiopia; 3 Department of Health Economics and Health Service Management, Bahir Dar University, Bahir Dar, Ethiopia; 4 Department of Adult Nursing, Bahir Dar University, Bahir Dar, Ethiopia; 5 Department of Nursing, GAMBY Medical and Business College, Bahir Dar, Ethiopia; 6 Epidemiology and Biostatistics, Institute of Public Health, College of Medicine and Health Science, University of Gondar, Gondar, Ethiopia; 7 Department of Epidemiology and Biostatistics, Bahir Dar University, Bahir Dar, Ethiopia; 8 Department of Biostatistics and Epidemiology, Bahir Dar University, Bahir Dar, Ethiopia; 9 Bahir Dar Health Science College, Bahir Dar city, Ethiopia; 10 School of public health, Woldia University, Woldia, Ethiopia; Manipal Academy of Higher Education, INDIA

## Abstract

**Background:**

Cancer is the second leading cause of death in the world. Knowing the cancer risk factors could help the policy-makers to design appropriate preventive and control strategies.

**Objective:**

To investigate the awareness regarding risk factors and determinants of cancers among Bahir Dar city residents, northwest, 2019

**Methods:**

A community-based cross-sectional study was employed. A systematic random sampling technique was carried out to select 845 study participants from May 1 to June 30, 2019. A validated structured cancer awareness measuring tool was used to collect the data. The data were entered into the Epi-data version 3.1 and analyzed using SPSS version 21 software. A simple logistic regression was run, and AOR (adjusted odds ratio) at a 95% confidence interval was used to identify the determinants of awareness regarding risk factors of cancers.

**Results:**

Nearly twenty percent of the respondents had a good awareness regarding risk factors of cancers. An orthodox Christian (AOR = 3. 2; 95%CI: 1.8, 5.6), college graduated (AOR = 2. 3; 95%CI:1.1, 4.9), a family member with cancer (AOR = 2. 0; 95%CI: 1.3, 3.3), and living in a rental house (AOR = 0. 6; 95%CI: 0.4, 0.9) were significantly associated with awareness regarding risk factors of cancers.

**Conclusion:**

The study revealed that awareness regarding risk factors of cancers was very low in the study area. Being Orthodox Christian, college graduated, a family member with cancer, and living in the rental house were the determinants of awareness regarding risk factors of cancers.

## Background

Noncommunicable diseases (NCDs) are currently responsible for the majority of global deaths [1]. Cancer is among the NCDs that creates a significant healthcare problem and a leading cause of death worldwide [2]. It is a large group of diseases that can affect any organ or tissue of the body when abnormal cells grow uncontrollably, go beyond their usual boundaries to invade adjoining parts of the body, and/or spread to other organs [3].

Cancer is the second leading cause of death globally and is estimated to account for 18.1 million new cases and 9.6 million deaths in 2018 [4]. The morbidity and mortality due to cancer are rapidly increasing throughout the world. The reasons are multifaceted but reflect both aging and growth of the population, as well as changes in the prevalence and distribution of the main risk factors for cancer [5]. Globally, cancer continues to exert tremendous physical, emotional, and financial strain on individuals, families, and health systems [6]. The burden is worsened in lower-income countries as the health systems are least prepared to manage this burden, and large numbers of cancer patients do not access timely diagnosis and treatment [7].

The majority of cancer-related deaths occur in low-and middle-income countries, most likely because of delayed clinical presentation of the disease [8]. Cancer in sub-Saharan Africa [9] is on the rise caused by rapid population growth, higher life expectancy, and the adoption of unhealthy lifestyles. In this region, cancer is becoming a critical challenge for health services due to the rising numbers of patients [3].

In Africa, cancer is an emerging public health issue with an estimate of 715,000 new cases and 542,000 deaths per year [9]. A 70% increment is expected in annual new cancer cases between 2012–2030. This is quicker than in any other region of the world [10].

Though the etiology of most cancers is not known, it has several risk factors. The risk factors vary based on geographic features and lifestyle-related habits of the residents. However, various studies revealed that tobacco smoking, alcohol consumption, genetics, obesity, physical inactivity, unhealthy diets, STDs, HIV, sun exposure, and exposure to carcinogens were the identified risk factors of cancer [11–18].

Increasing awareness of cancer risk factors can help the community-members to enhance cancer-screening behavior and to exercise appropriate cancer preventive-practice [11]. Determining the awareness level of the residents regarding cancer risk factors is a critical element to try to help policymakers carry out evidence-based interventions and institute community-based screening programs [19, 20].

Though several studies were carried out in different parts of the country, no research has been conducted in this study area. Therefore, we aimed to determine the level of awareness regarding risk factors and the determinants of cancers among Bahir Dar city residents.

## Methods

### Study design, setting, and period

A community-based cross-sectional study was conducted among Bahir Dar city residents from May 1 to June 30, 2019. Bahir Dar city is the capital of the Amhara Regional state, which is 565 Km from Addis Ababa, the capital city of Ethiopia. The city administratively divides into six sub-cities. The total population of the city is estimated to be 249,851. Of these, 125,425 were males [21]. In the city, there are five hospitals (two Public and three Private), eight public health centers, and six private clinics.

### Population

All adults residing in the sub-cities of Bahir Dar city were the source population; and adults living in the four selected sub-cities of Bahir Dar city were included for the study.

### Sample size determination and sampling procedure

The sample size calculated using a single population proportion formula with the assumption of a 95% confidence interval, a 5% margin of error, and a 50% awareness regarding risk factors of cancers. Finally, a design effect of 2 and a non-response rate of 10% was considered, yielding a total sample of 845.

A multistage sampling technique was used to recruit the study participants. At the first stage, four out of six sub-cities (Hidar 11, Fasilo, Belay zeleke, and Tana) were selected by a simple random sampling method. Then, from each sub-city, we took the list of adults (whose age is ≥ 18 years of age) from the city administration office. A systematic random sampling technique was employed to select households from each sub-city. Each adult of 18 years of age or older were interviewed from each selected household. When more than one eligible adult is found in each selected household, the lottery method was employed. However, mentally ill and severely sick adults were excluded from the study.

### Data collection process

The pre-tested, interviewer-administered, structured Amharic questionnaire was used to collect the data (**S1 File**). This tool is a standardized and validated data collection tool for awareness regarding risk factors of cancers. The questionnaire had two components: socio-demographic aspects and questions for assessing awareness regarding risk factors of cancers. All the enumerators collected the data after having written consent from participants. To assure the quality of the data, and to make sure that all team members were able to administer the questionnaires uniformly, three-day of rigorous training was given to six nurses with bachelor’s degree (data collectors) and to one supervisor with master’s degree.

Data collectors and the supervisor carried out role-plays and then had field pre-test activities in five percent of the total sample size before the actual data collection. At the end of every data collection day, the supervisor examined each questionnaire and gave pertinent feedback to the data collectors. The internal consistency (Cronbach alpha) level of the pretest of awareness regarding risk factors of cancers was 0.79.

### Operational definition

Those adults who scored more than the mean value for the questions on the awareness regarding risk factors of cancers were considered as having a good awareness regarding risk factors of cancers. Otherwise, they were labelled as having poor awareness regarding risk factors of cancers.

### Data processing and analysis

The PI checked the collected data for completeness and consistency daily. The data were then entered into Epi Data version 3.1 software and exported to SPSS version 21 for analysis. Descriptive statistics were computed, and a simple Logistic regression model was used to identify the association between explanatory and outcome variables. Adjusted Odds ratio with 95% CI was used to measure the strength of association between explanatory variables and the awareness regarding risk factors of cancers. The model fitness checked using Hosmer and Lemeshow goodness of fit (P > 0.05). A p-value ≤ 0.2 at bivariate analysis was considered for variables to include in multivariable logistic regression analysis. Variables with a p-value of < 0.05 at multivariate analysis were considered as statistically significant predictors of awareness regarding risk factors of cancers.

### Ethics approval and consent to participate

Ethical approval was obtained from GAMBY Medical and Business College, Research, and health institute. The support letter was obtained from the Bahir Dar city administration. The objective of the study was clarified to the study participants, and they were also notified that they have the right to opt-out of the study at any point in the interview. Then, the written consent was secured from the respondents. The written consent and the data collection tools were documented and kept confidential in a secure place.

## Results

### Socio-demographic characteristics of the study participants

A total of 845 participants furnished data for the final analysis, with a response rate of 100%. The mean age of the participants was 29.8 (±10.4) years; About one-third (32.9%) of the respondents had completed secondary education. About 53.8% of the respondents were married, and 29.5% of the respondents lived in a rental house. Nearly 13.5% of the respondents had a family member living with cancer. Twenty percent of the respondents go to blessed places for holy water when they get sick (Table 1).

**Table 1:** Socio-demographic characteristics of the study participants in Bahir Dar city, northwest Ethiopia (n = 845).

Variable	Category	Frequency	Percentage
Age	18–29 years	499	59.1
	30–44 years	251	29.7
	≥ 45 years	95	11.2
Sex	Male	313	37.0
	Female	532	63.0
Religion	Orthodox	677	80.1
	Non-Orthodox	168	19.9
Marital status	Married	455	53.8
	Unmarried	390	46.2
Educational status	Unable to read and write	97	11.5
	Able to read and write	69	8.1
	Primary school	129	15.3
	Secondary school	278	32.9
	College and above	272	32.2
House arrangement	Private house	270	32.0
	Government house	88	10.4
	Rental house	249	29.5
	Living with family/friend	238	27.9
Occupational status	Private Employee	208	24.6
	Government Employee	146	17.3
	Merchant	106	12.5
	Student	201	23.8
	Housewife	89	10.5
	Unemployed	80	9.5
	Retired	15	1.8
Your family members had cancer	Yes	114	13.5
	No	731	86.5
Who had cancer	Me	3	2.6
	My partner	17	14.9
	Close family member	94	82.5
The decision you made when you got sick	Private health facility	285	33.7
	Public health facility	354	41.9
	Holy water	169	20.0
	I didn’t go anywhere	37	4.4

### The level of awareness regarding risk factors of cancers

The study revealed that one-hundred-seventy-one (20.2%) of the study participants had good awareness regarding risk factors of cancers (Table 2).

**Table 2:** Awareness regarding risk factors of cancers in the study participants of Bahir Dar city, northwest Ethiopia 2019, (n = 845).

Variables	Category	Frequency	Percent
Unexplained bleeding could be a sign of cancer	Yes	412	48.8
	No	224	26.5
	Don’t know	209	24.7
A persistent cough or hoarseness could be a sign of cancer	Yes	150	17.8
	No	491	58.1
	Don’t know	204	24.1
A persistent change in bowel or bladder habits could be a sign of cancer	Yes	109	12.9
	No	493	58.3
	Don’t know	243	28.8
A persistent difficulty swallowing could be a sign of cancer	Yes	137	16.2
	No	467	55.3
	Don’t know	241	28.5
A change in the appearance of a mole could be a sign of Cancer	Yes	114	13.5
	No	539	63.8
	Don’t know	192	22.7
A sore that does not heal could be a sign of cancer	Yes	432	51.1
	No	248	29.3
	Don’t know	165	19.6
An unexplained lump or swelling could be a sign of cancer	Yes	468	55.4
	No	214	25.3
	Don’t know	163	19.3
Persistent unexplained pain could be a sign of cancer	Yes	319	37.8
	No	280	33.1
	Don’t know	246	29.1
Unexplained weight loss could be a sign of cancer	Yes	208	24.6
	No	401	47.5
	Don’t know	236	27.9
Smoking cigarettes can increase a person’s chance of developing cancer	Yes	633	74.9
	No	153	18.1
	Don’t know	59	7.0
Exposure to another person’s cigarette smoke can increase a person’s chance of developing cancer	Yes	479	56.7
	No	233	27.6
	Don’t know	133	15.7
Drinking more than 1 unit of alcohol a day can increase a person’s chance of developing cancer	Yes	323	38.2
	No	332	39.3
	Don’t know	190	22.5
Eating less than 5 portions of fruit and vegetables a day can increase a person’s chance of developing cancer	Yes	182	21.5
	No	458	54.2
	Don’t know	205	24.3
Eating red or processed meat once or more /day can increase a person’s chance of developing cancer	Yes	245	29.0
	No	336	39.8
	Don’t know	264	31.2
Being overweight (BMI over 25) can increase a person’s chance of developing cancer	Yes	195	23.1
	No	312	36.9
	Don’t know	338	40
Getting sun burnt more than once as a child can increase a person’s chance of developing cancer	Yes	152	18.0
	No	356	42.1
	Don’t know	337	39.9
Being over 70 years old can increase a person’s chance of developing cancer	Yes	260	30.8
	No	424	50.2
	Don’t know	161	19.0
Having a close relative with cancer can increase a person’s chance of developing cancer	Yes	166	19.7
	No	466	55.1
	Don’t know	213	25.2
**The composite score of awareness regarding risk factors of cancers**	**Good**	**171**	**20.2**
	**Poor**	**674**	**79.8**

### The simple logistic regression analysis on awareness regarding risk factors of cancers

In the univariate logistic regression analysis, religion, level of education, house arrangement, and a family member with cancer were the factors associated with awareness regarding risk factors of cancers at a 20% level of significance. In the multivariable logistic regression analysis, religion, level of education, house arrangement, and a family member with cancer were predictors of awareness regarding risk factors of cancers at a p-value of less than < 0.05. Similarly, for those participants who are Orthodox Christianity followers, the odds of awareness regarding risk factors of cancers were 3.2 (AOR = 3. 2; 95%CI: 1.8, 5.6) times higher compared to other religious followers. For those respondents who are college graduated, the odds of awareness regarding risk factors of cancers were 2.3 (AOR = 2. 3; 95%CI:1.1, 4.9) times more compared with unable to read and write. For the participants, who were living in the rental house, the odds of awareness regarding risk factors of cancers were 0.6 (AOR = 0. 6, 95% CI: 0.4, 0.9) times lower than compared with those who had their own house. Moreover, for those participants whose family members had cancer, the odds of awareness regarding risk factors of cancers were two (AOR = 2. 0, 95% CI: 1.3, 3.3) times higher compared with those who had no family members with cancer (Table 3).

**Table 3:** Simple logistic regression analysis on factors of awareness regarding risk factors of cancers in Bahir Dar city residents, northwest Ethiopia, 2019 (n = 845).

Variable		Awareness regarding risk factors of cancers		COR (95%CI)	AOR (95%CI)	P-value
		Good	Poor			
Religion	Orthodox	154	523	2.6 (1.5, 4.5)	**3.2 (1.8, 5.6)**	**0.001**
	Non-Orthodox*	17	151	1.00	1.00	
Educational status	Unable to read and write	10	59	1.00	1.00	
	Able to read and write only	18	79	1.34 (0.6, 3.1)	1.3 (0.5, 3.1)	0.57
	Primary school	21	108	1.15 (0.5, 2.6)	1.2 (0.5, 2.8)	0.71
	Secondary school	46	232	1.2 (0.6, 2.5)	1.3 (0.6, 2.8)	0.53
	College graduated	76	196	2.3 (1.1, 4.7)	**2.3 (1.1, 4.9)**	**0.03**
House arrangement	Private house	58	212	1.00	1.00	
	Government house	27	61	1.6 (0.9, 2.8)	1.5 (0.8, 2.7)	0.23
	Rental house	36	213	0.6 (0.4, 0.9)	**0.6 (0.4, 0.9)**	**0.02**
	Living with family	50	188	0.9 (0.6, 1.5)	0.9 (0.6, 1.5)	0.85
A family member with cancer	Yes	38	76	2.2 (1.5, 3.5)	**2.0 (1.3, 3.3)**	**0.004**
	No	133	598	1.00	100	

## Discussion

In Ethiopia, the early detection and control programs of cancer rely on a community’s awareness regarding risk factors of cancers [22]. A community’s awareness regarding risk factors of cancers helps individuals to avoid unhealthy lifestyles [22]. These unhealthy lifestyles are modifiable and are related to what we eat, how we exercise, and which drugs, medications, and supplements we take [23]. The present study determined the level of good awareness regarding risk factors of cancers among Bahir Dar city residents, which was 20.2%. This finding was very low as compared to the study findings of Northern Tanzania, 90% [22], and 36.8%, Oman, Western Asia [24]. The possible explanation for this discrepancy might be the difference in study settings, social norms, lifestyles, health care set up, and health information dissemination. The current study also identified the factors affecting good awareness regarding risk factors of cancers in Bahir Dar city. Being Orthodox Christian, college graduated, had a family member with cancer, and living in the rental house were the predictors of awareness regarding risk factors of cancers.

For orthodox Christianity, the odds of awareness of cancer were 3.2 times higher compared with the non-Orthodox Christianity religion followers. This could be the Orthodox Christian followers mostly spent their time by fasting (without eating food) and could protect them from eating/drinking carcinogenic diets.

Likewise, for those college graduated respondents, the odds of good awareness regarding risk factors of cancers were 2.3 (AOR = 2. 3; 95%CI:1.1, 4.9) times more compared with unable to read and write. Thus, people with higher educational levels were more likely to have better awareness regarding risk factors of cancers that can influence the risks of getting cancer. This is due to the educated adults have the possibility of utilizing different social media, including internet access. Using different media might allow them to have wider health information dissemination, which enhances their awareness regarding risk factors of cancers.

Family members with cancer were the other determinant factor that affects the awareness regarding risk factors of cancers of adults. For those participants whose family members had cancer, the odds of good awareness regarding risk factors of cancers were two (AOR = 2. 0, 95% CI: 1.3, 3.3) times higher compared with those who had not. This finding was supported by the study findings of Iran [25] and Saudi Arabia [26]. The possible justification for this could be, once the family member got cancer, its members would be afraid of the complication of the cancer ill family member. And they have the tendency of getting information on the risk factors of cancer, which would allow them to have better awareness regarding risk factors of cancers.

Moreover, our study also identified those adults who are living in a rental house were 0.6 (AOR = 0. 6, 95% CI: 0.4, 0.9) times less likely to associate with a good awareness regarding risk factors of cancers compared with those who had their own house.

The current study is also supported by study findings of Denmark [27], Japan [28], and Veterans [29]. This might be people with low socio-economic status is generally less likely to aware of regarding risk factors of cancers. In other words, higher socio-economic and demographic status is strongly associated with greater awareness regarding risk factors of cancers. Higher socio-economic and demographic status is strongly associated with greater awareness regarding risk factors of cancers [30–33].

### Strength of the study

- As this is a community-based work with an adequate sample its finding is highly inferable for the city.
- Participants who had poor knowledge were advised to visit the nearby health institution for further cancer-related information and communication.
- The study involved both males and females, and it could show the overall cancer awareness level of respondents.

### Limitations of the study

- Since the study was cross-sectional, it is difficult to ascertain the association of the identified factors and cancer awareness.

## Conclusions

The level of awareness regarding risk factors of cancers in Bahir Dar city residents is low as per the national standard. The study revealed that only 20.2% of respondents had good awareness regarding risk factors of cancers. Religion, level of education, house arrangement, and a family member who had cancer were predictors of awareness regarding risk factors of cancers. It is advisable to give due attention to creating awareness regarding risk factors of cancers to the community members by religious leaders, mass media, and the health care workers to enhance the communities understanding of cancer; and the residents to raise their hand in the prevention and control strategies of current government efforts.

## Supporting information

S1 File (PDF)

S2 File (SAV)
