# Supplementary material for: Awareness regarding risk factors and determinants of cancers among Bahir Dar city residents, Northwest Ethiopia
Source: PLoS One. 2021 Apr 23;16(4):e0248520. doi: 10.1371/journal.pone.0248520 (PMC8064596; doi:10.1371/journal.pone.0248520)
Supplement: S1 File — (PDF) [file pone.0248520.s001.pdf]

English Questionnaire

PART-1: Sociodemographic characteristics of the respondents

S. No	**Variables**	**Categories**	**Skip**
101	Age	---------------years	
102	Sex	1. Male<br>2. Female	
103	Religion	1. Orthodox<br>2. Muslim<br>3. Protestant<br>4. Catholic<br>5. Others (if any, specify)________	
104	Ethnicity	1. Amhara<br>2. Oromo<br>3. Tigrie<br>4. Agew<br>5. Others (if any, specify) ________	
105	Marital status	1. Married<br>2. Unmarried<br>3. Widowed<br>4. Divorced	
106	Educational status	1. Unable to read and write<br>2. Able to read and write<br>3. Primary school<br>4. Secondary school<br>5. College and above	
107	House arrangement	1. Private house<br>2. Government house<br>3. Rental house<br>4. Living with family/friend	

108	Occupational status	1. Private Employee<br>2. Government Employee<br>3. Merchant<br>4. Student<br>5. Housewife<br>6. Unemployed<br>7. Retired	
109	Your family members had cancer	1. Yes<br>2. No	
110	Who had cancer	1. Me<br>2. My partner<br>3. Close family member	
111	The decision you made when you got sick	1. Private health facility<br>2. Public health facility<br>3. Holy water<br>4. I didn't go anywhere	

PART-2: Awareness of cancer risk factors related characteristics of the respondents

S. No	**Variables**	**Categories**	**Skip**
201	Unexplained bleeding could be a sign of cancer	1. Yes<br>2. No<br>3. Don't know	
202	A persistent cough or hoarseness could be a sign of cancer	1. Yes<br>2. No<br>3. Don't know	
203	A persistent change in bowel or bladder habits could be a sign of cancer	1. Yes<br>2. No<br>3. Don't know	
204	A persistent difficulty swallowing could be a sign of cancer	1. Yes<br>2. No	

		3. Don’t know	
205	A change in the appearance of a mole could be a sign of Cancer	1. Yes<br>2. No<br>3. Don’t know	
206	A sore that does not heal could be a sign of cancer	1. Yes<br>2. No<br>3. Don’t know	
207	An unexplained lump or swelling could be a sign of cancer	1. Yes<br>2. No<br>3. Don’t know	
208	Persistent unexplained pain could be a sign of cancer	1. Yes<br>2. No<br>3. Don’t know	
209	Unexplained weight loss could be a sign of cancer	1. Yes<br>2. No<br>3. Don’t know	
210	Smoking cigarettes can increase a person’s chance of developing cancer	1. Yes<br>2. No<br>3. Don’t know	
211	Exposure to another person’s cigarette smoke can increase a person’s chance of developing cancer	1. Yes<br>2. No<br>3. Don’t know	
212	Drinking more than 1 unit of alcohol a day can increase a person’s chance of developing cancer	1. Yes<br>2. No<br>3. Don’t know	
213	Eating less than 5 portions of fruit and vegetables a day can increase a person’s chance of developing cancer	1. Yes<br>2. No<br>3. Don’t know	
214	Eating red or processed meat once or more /day can increase a person’s chance of developing cancer	1. Yes<br>2. No<br>3. Don’t know	

215	Being overweight (BMI over 25) can increase a person's chance of developing cancer	1. Yes<br>2. No<br>3. Don't know	
V 216	Getting sun burnt more than once as a child can increase a person's chance of developing cancer	1. Yes<br>2. No<br>3. Don't know	
217	Being over 70 years old can increase a person's chance of developing cancer	1. Yes<br>2. No<br>3. Don't know	
218	Having a close relative with cancer can increase a person's chance of developing cancer	1. Yes<br>2. No<br>3. Don't know	

Thank you very much for your participation!!
